# Safety and short-term efficacy of neoadjuvant FLOT therapy in cisplatin-unfit patients with resectable esophageal squamous cell carcinoma

**DOI:** 10.1007/s10388-025-01160-5

**Published:** 2025-10-14

**Authors:** Yuri Yoshinami, Shun Yamamoto, Kazuhiro Shiraishi, Hiroshi Imazeki, Kazuki Yokoyama, Yoshitaka Honma, Daisuke Kurita, Koshiro Ishiyama, Junya Oguma, Taiki Hashimoto, Tairo Kashihara, Hiroyuki Daiko, Ken Kato

**Affiliations:** 1 Department of Head and Neck, Esophageal Medical Oncology, National Cancer Center Hospital, 5-1-1 Tsukiji, Chuo-ku, Tokyo, 104-0045 Japan; 2 Department of Esophageal Surgery, National Cancer Center Hospital, Tokyo, Japan; 3 Department of Diagnostic Pathology, National Cancer Center Hospital, Tokyo, Japan; 4 Department of Radiation Oncology, National Cancer Center Hospital, Tokyo, Japan

**Keywords:** Esophageal, Squamous cell carcinoma, Neoadjuvant chemotherapy, FLOT

## Abstract

**Backgrounds:**

The standard neoadjuvant therapy for resectable locally advanced esophageal squamous cell carcinoma (LA-ESCC) is a combination of docetaxel, cisplatin (CDDP), and 5-fluorouracil in Japan. However, patients with renal or cardiac dysfunction and elderly patients were unfit for CDDP-containing regimens due to toxicity.

In this context, 5-fluorouracil and leucovorin, oxaliplatin, docetaxel (FLOT) therapy, which is the standard neoadjuvant therapy for esophagogastric adenocarcinoma in Western countries, offers an alternative that can be administered to the patients who are unfit for CDDP. However, the safety and short-term efficacy of neoadjuvant FLOT therapy in patients with LA-ESCC remain unclear.

**Materials and methods:**

This retrospective study analyzed patients with resectable LA-ESCC who received neoadjuvant FLOT from February 2021 to December 2023. Four cycles of FLOT were administered every 2 weeks, and then the subjects underwent esophagectomy. Adverse events were evaluated according to the CTCAE version 5.0, and pathological response and survival outcomes were evaluated for efficacy.

**Results:**

Forty-six patients were included in this study. Median age was 76 years (range 57–84 years). Clinical stage III and IVB were the most frequent, at 61% and 20%, respectively. During the neoadjuvant therapy, the most common grade 3 or higher adverse events were neutropenia (65%) and leukopenia (50%). Of 36 patients who underwent surgery, pathologic complete response (ypT0N0) was observed in 5 patients (13.9%). The median progression-free survival and overall survival were 15.0 and 25.2 months, respectively.

**Conclusions:**

Neoadjuvant FLOT demonstrated manageable safety profiles and promising efficacy in patients with resectable LA-ESCC who were CDDP-unfit.

## Introduction

Esophageal cancer (EC) is the seventh most common cancer worldwide and the sixth leading cause of cancer-related death [1]. Histologically, EC can be divided into two main types: squamous cell carcinoma and adenocarcinoma. In Western countries, adenocarcinoma accounts for more than 60% of esophageal cancers, while in Asia, East Africa, and South Africa, squamous cell carcinoma accounts for more than 80%.

In the Western countries, neoadjuvant chemoradiotherapy (neo CRT) is frequently used as standard treatment mainly for resectable locally advanced esophageal squamous cell carcinoma (LA-ESCC), since a randomized controlled trial comparing neo CRT followed by surgery with surgery alone (CROSS trial) demonstrated superiority in overall survival (OS) [2]. This was a randomized controlled trial that assigned patients with resectable esophageal and esophagogastric junction cancer (n = 368) to receive either surgery alone or neo CRT (5 weeks of carboplatin plus paclitaxel and 41.4 Gy/23 fr of radiation therapy) plus surgery. However, the ESOPEC trial recently showed that neoadjuvant chemotherapy was superior to neo CRT [3]. The ESOPEC trial was a multicenter, prospective, randomized trial comparing a group of patients with resectable locally advanced esophageal adenocarcinoma who received the CROSS regimen followed by surgery as neoadjuvant therapy, and a group who received 5-FU plus leucovorin, oxaliplatin, and docetaxel (FLOT) therapy administered pre- and post-surgery followed by surgery. CROSS is administered over 5 weeks in 5 cycles, while FLOT is administered over 8 weeks in 4 cycles every 2 weeks. After surgery, the FLOT group receives FLOT over 8 weeks in 4 cycles every 2 weeks. Furthermore, the NeoRes study compared neoadjuvant chemotherapy with neo CRT and found no additional benefit of neo CRT on survival [4]. This was a multicenter, prospective, randomized trial comparing chemotherapy alone with neo CRT in patients with resectable esophageal cancer or gastroesophageal junction cancer. The chemotherapy group underwent 3 cycles of cisplatin plus 5-FU every 3 weeks followed by surgery, while the neo CRT group underwent cisplatin plus 5-FU combined with 40 Gy/20 fractions of radiation therapy followed by surgery. Also, based on the results of the CheckMate 577 trial, the Japanese guideline strongly recommends one year of adjuvant nivolumab for patients with locally advanced esophageal cancer who have undergone R0 resection without a pCR after neoadjuvant CRT [5].

In Japan, neoadjuvant docetaxel, cisplatin (CDDP), and 5-fluorouracil (DCF) followed by surgery is the standard of care based on the results of the JCOG1109 trial [6]. Nevertheless, neoadjuvant DCF (neo DCF) is more toxic than CF, and neoadjuvant CF remains a treatment option, especially in elderly and frail patients [7]. Furthermore, CDDP is highly nephrotoxic and requires high volume hydration, making it unfit for treatment including CDDP in patients with renal or cardiac dysfunction. In clinical practice, for patients with resectable LA-ESCC who are unfit for CDDP, neoadjuvant chemotherapy (nedaplatin + 5-fluorouracil (FN), oxaliplatin + leucovorin + 5-fluorouracil (FOLFOX), DCF with dose reduction) followed by surgery [8, 9]. FOLFOX and FN can be used in patients with CDDP intolerance, but because they are two-drug combinations, the response rate is only about 30–40%. On the other hand, FLOT can be expected to achieve a response rate of 50–60%, and it may be possible to replace DCF in more advanced cases. However, the evidence for these approaches is limited, with reports based solely on small retrospective studies, and no standard treatment has been established.

On the other hand, FLOT therapy is the standard neoadjuvant treatment for resectable locally advanced esophagogastric adenocarcinoma (LA-EGA) in the Western countries based on the results of the FLOT4 [10] and ESOPEC[3] trials; FLOT is also less gastrointestinal and nephrotoxic than regimens that include CDDP and do not require rehydration [10]. However, these trials have focused on adenocarcinomas, and there are few reports on the safety and efficacy of neoadjuvant FLOT (neo FLOT) in LA-ESCC patients. Furthermore, in squamous cell carcinoma, the JCOG9907 trial demonstrated that neoadjuvant therapy was superior to adjuvant therapy, leading to the development of neoadjuvant therapy in Japan. FLOT has been established as an option to DCF in JCOG1109, and therefore adjuvant therapy is not routinely performed in Japan. On the other hand, though there was no clear scientific evidence and the Japanese guideline [11] did not define the level of recommendation for adjuvant nivolumab use after neo-DCF, many doctors have adopted adjuvant nivolumab in their clinical practice. While its value remains an open clinical question, a randomized controlled trial is currently underway to compare postoperative observation, postoperative nivolumab, and postoperative S-1 treatment [12].

This study evaluated the safety and efficacy of neo FLOT in CDDP-unfit patients with resectable LA-ESCC.

## Methods

### Patients

This retrospective study analyzed LA-ESCC patients who received neo FLOT at the National Cancer Center Hospital from February 2021 to December 2023. The main selection criteria were as follows: histologically diagnosed esophageal squamous cell carcinoma; cT1N1-3M0, cT2-3N0-3M0, or cT1-3N0-3M1 (M1 confined to resectable supraclavicular lymph node metastases) according to the International Union of Cancer Classification of Malignancy TNM, 8th edition; and no prior treatment for EC. The clinical staging was evaluated using CT and endoscopy. The reasons for selecting FLOT were categorized as decreased renal function, decreased cardiac function, advanced age, or weakness. In cases where it was difficult to make a retrospective judgment based on the medical records, the reason was recorded as the physician’s determination. In addition, “CDDP-unfit” includes patients who require a reduction in cisplatin.

This study was approved by the Institutional Review Board of the National Cancer Center Hospital (Approval No. 2020-287).

### Treatment

Neo FLOT (5-FU 2600 mg/m^2^ day1-2 over 24 h, leucovorin 200 mg/m^2^ day1, oxaliplatin 85 mg/m^2^ day1, docetaxel 50 mg/m^2^ day1) was administered intravenously every 2 weeks for 4 cycles. Granulocyte colony-stimulating factor (G-CSF) was used as primary prophylaxis on days 7–14 of each course, at the discretion of the attending physician. Also, 4 to 8 weeks after the end of FLOT, surgery was performed in all patients who were eligible for surgery.

### Assessments and statistics

Computed tomography (CT) scans were performed before the start of neo FLOT, after the cycle 2, and before surgery. Laboratory tests were performed at the beginning of each treatment cycle and before surgery. The patients’ general condition was assessed using the Performance Status (PS) created by the Eastern Cooperative Oncology Group (ECOG).

The primary endpoints of this study were adverse events and pathological response of neo FLOT therapy. Adverse events were evaluated according to the Common Terminology Criteria of Adverse Events (CTCAE) version 5.0. Pathological response of primary lesion was classified according to the percentage of tumor tissue using the grading system based on the Japanese Classification of Esophageal Cancer, 12th edition [13]: grade 0, no part of tumor affected; grade 1a, very slightly effective; grade 1b, slightly effective; grade 2, moderately effective; and grade 3, markedly effective. For pathological T0 or Tis N1-3, T0N1/TisN1 was defined as Stage IIB, T0N2/TisN2 as Stage III, and T0N3/TisN3 as Stage IVA. Postoperative complications were evaluated using the Clavien-Dindo classification, with the evaluation period being from surgery to 28 days.

Additionally, to assess efficacy, the response rate to neoadjuvant chemotherapy, complete resection rate (R0), progression-free survival (PFS), and overall survival (OS) were used. Response to chemotherapy was assessed according to the Response Evaluation Criteria in Solid Tumors (RECIST) version 1.1 [14]. PFS was defined as the time from the start of neo FLOT therapy to the disease progression, recurrence after surgery or death. Additionally, for patients who did not undergo surgery, PFS was defined as the time to recurrence, progression, or death following subsequent treatment. OS was defined as the time from the start of neo FLOT therapy to death or the time censored for patients who were alive at the last follow-up. Survival was estimated using Kaplan–Meier curves.

## Results

### Patients and treatment

**Fig. 1:**
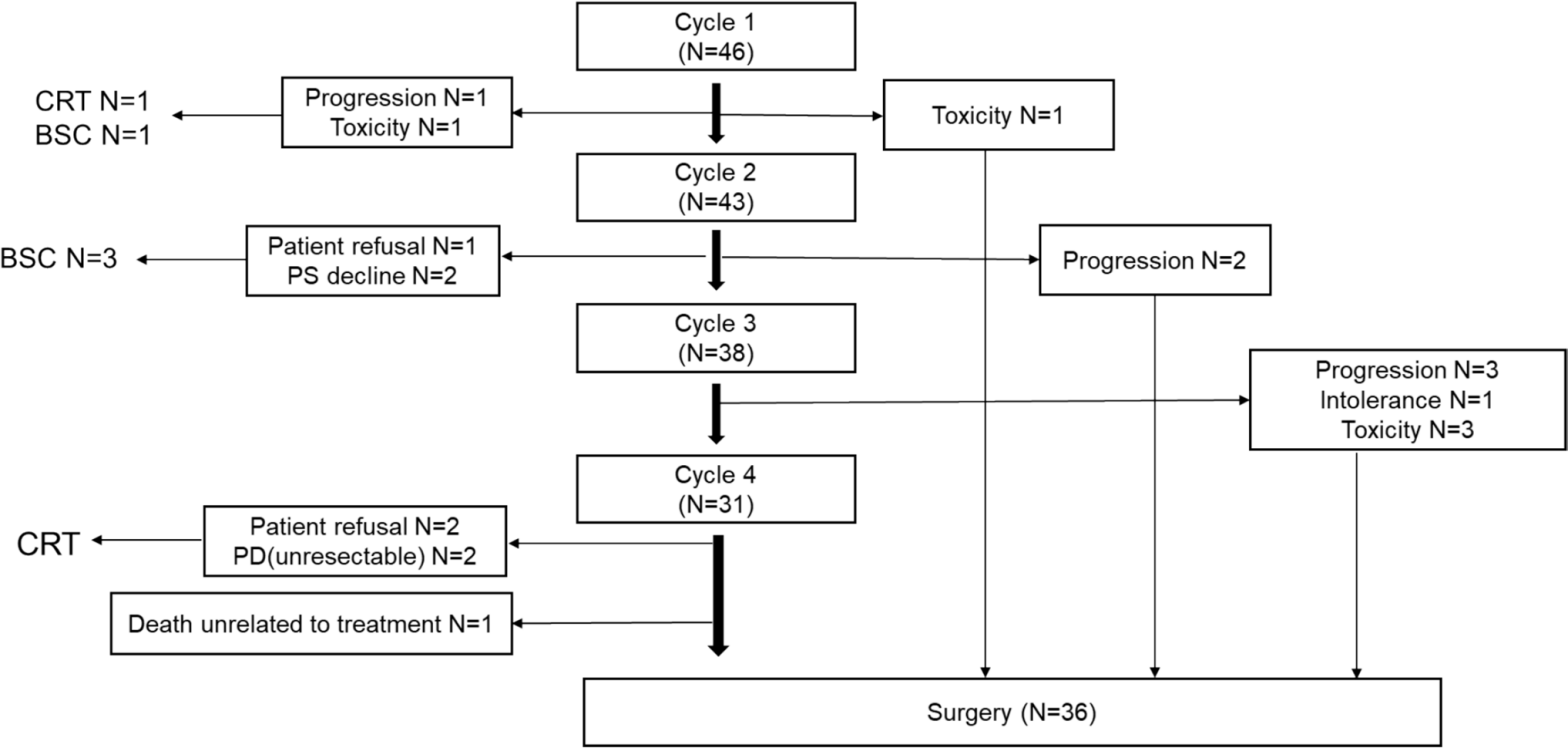
Treatment flow for all patients who were received neoadjuvant FLOT therapy. CRT: chemoradiotherapy, BSC: best supportive care, PS: performance status, PD: progressive disease

Forty-six patients received neo FLOT therapy during the study period. Characteristics of study participants at the start of chemotherapy are shown in Table 1. Median age was 76 years (range 57–84). ECOG-PS was 0 for 39% and 1 for 59%. Treatment details are shown in Fig. 1. The reasons for selecting FLOT were: 14 cases (30.4%) had decreased renal function, 10 cases (21.7%) were elderly or frail, 3 cases (7.5%) had decreased cardiac function, and 19 cases (41.3%) were determined by the physicians. Regarding TNM stage, there were 6 people in T1, 4 people in T2, and 36 people in T3, and regarding N, there were 5 people in N0, 22 people in N1, 16 people in N2, and 3 people in N3. There were 11 people in M1, but all of them had only resectable supraclavicular lymph node metastasis.

**Table 1:** Characteristics of patients

	N = 46
Median age (Range)	76 (57–84)
Gender (%)	
Male/Female	37 (80)/9 (20)
ECOG PS (%)	
0/1–2	18 (39)/29 (61)
Clinical T stage (%)	
T1/T2/T3	6 (13)/4 (9)/36 (78)
Clinical N stage (%)	
N0/N1/N2/N3	5 (11)/22 (48)/16 (35)/3 (7)
Clinical M stage (%)	
M0/M1	37 (80)/9 (20)
Clinical stage (%)	
I	5 (11)/3 (7)/28 (61)/1 (2)/9 (20)
Tumor location (%)	
Ce/Ut/Mt/Lt	2(4)/5(11)/25(54)/14(30)
Histology (%)	
Squamous cell carcinoma	46 (100)

*ECOG PS* Eastern Cooperative Oncology Group performance status, *FLOT* 5-fluorouracil, leucovorin, oxaliplatin, and docetaxel, *TNM* tumor node metastasis, *Ce* cervical, *Ut* upper thoracic esophagus, *Mt* middle thoracic esophagus, *Lt* lower thoracic esophagus

Forty-six patients received one cycle of neoadjuvant chemotherapy, and 31 were able to complete it. Three patients discontinued after one cycle due to grade 3 febrile neutropenia (n = 2) and progressive disease (n = 1). Five patients discontinued after the second course due to patient preference (n = 1), worsening PS (n = 2) and progressive disease (n = 2). Seven patients discontinued after the third course due to decreased renal function (n = 1), grade 3 skin rash (n = 2), grade 3 pneumonia (n = 1), and progressive disease (n = 3). Thirty-one patients completed four cycles, but five did not undergo surgery (two due to patient preference, two due to progressive disease, and one due to an unknown cause of death). Finally, 36 patients (78.2%) underwent esophagectomy.

### Safety of neo FLOT therapy

Adverse events during neo FLOT therapy were summarized in Table 2. The most common grade ≥3 hematologic toxicities were neutropenia in 30 patients (65.2%) and leukopenia in 23 patients (50.0%). Febrile neutropenia (FN) occurred in 5 patients (10.9%). G-CSF was used prophylactically during treatment in 35 patients (76.1%). The characteristics of cases in which G-CSF was used prophylactically were similar to those of the overall population. The most common grade 3 non-hematologic toxicities were anorexia in 2 patients (4.3%), nausea in 1 patient (2.2%), and AST/ALT elevation in 1 patient (2.2%), and skin rash in 1 patient (2.2%). There were no Grade 4 non-hematological toxicities.

**Table 2:** Adverse events during neoadjuvant chemotherapy

	N = 46					
	G1	G2	G3	G4	≥G3	Any grade
Hematologic toxicity (%)						
Leukopenia	2 (4)	16 (35)	21 (46)	2 (4)	23 (50)	42 (91)
Neutropenia	1 (2)	13 (28)	8 (17)	22 (48)	30 (65)	44 (96)
Anemia	32 (70)	10 (22)	1 (2)	0 (0)	1 (2)	43 (93)
Thrombocytopenia	13 (28)	3 (7)	2 (4)	0 (0)	2 (4)	18 (39)
Non-hematologic toxicity (%)						
Nausea	10 (22)	3 (7)	1 (2)	0 (0)	1 (2)	14 (30)
Anorexia	13 (28)	5 (11)	2 (4)	0 (0)	2 (4)	20 (43)
Mucositis	7 (15)	2 (4)	0 (0)	0 (0)	0 (0)	9 (20)
Malaise	22 (48)	0 (0)	0 (0)	0 (0)	0 (0)	22 (48)
Peripheral neuropathy	13 (28)	0 (0)	0 (0)	0 (0)	0 (0)	13 (28)
AST increased	2 (4)	0 (0)	1 (2)	0 (0)	1 (2)	3 (7)
ALT increased	2 (4)	0 (0)	1 (2)	0 (0)	1 (2)	3 (7)
Hyponatremia	21 (46)	1 (2)	0 (0)	0 (0)	0 (0)	22 (48)
Febrile neutropenia	–	–	5 (11)	0 (0)	5 (11)	5 (11)
Rash maculo-papular	1 (2)	2 (4)	1 (2)	0 (0)	1 (2)	4 (9)

Based on the attending physician’s judgment, 7 patients (15.2%) required dose reduction from the first cycle due to renal dysfunction, all with creatinine clearance ≤40 mL/min (Cockcroft–Gault). Dose reduction after the first cycle was required in 9 patients (19.6%) as determined by the attending physician: neutropenia in 5 patients (10.9%), anorexia in 2 patients (4.3%), febrile neutropenia in 1 patient (2.2%), and acute kidney injury in 1 patient (2.2%). Of the total, dose reduction was performed in 22 patients (47.8%), most commonly for neutropenia. Malaise and anorexia were the next most common reasons (each in 2 patients, 4.3%).

### Efficacy and surgical outcomes

**Fig. 2:**
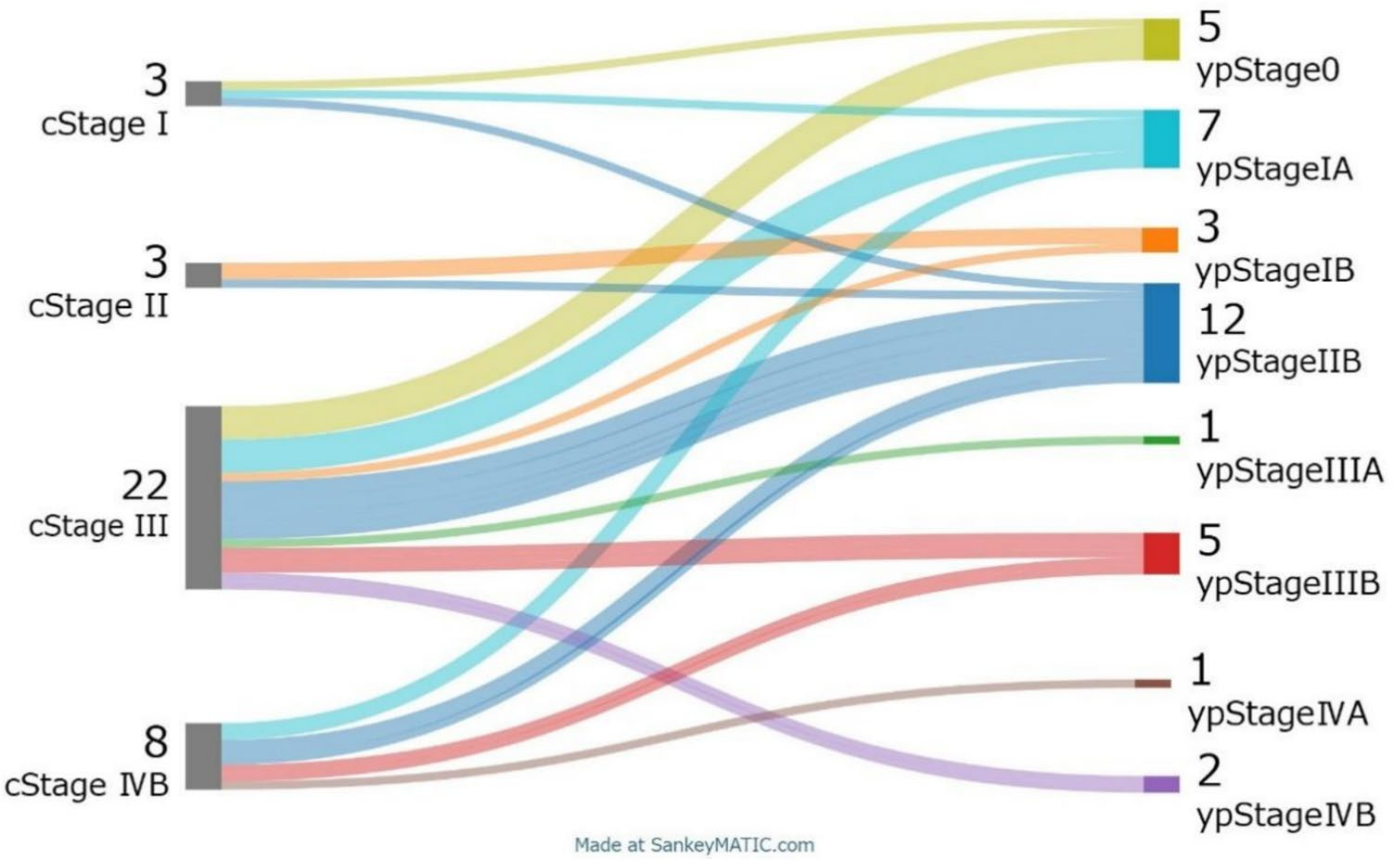
Relationship between clinical stage and pathological stage in 36 patients. Pathological T0-is N+ was treated as pT1 N+. Downstaging was possible in 72% patients

According to preoperative RECIST v1.1 evaluation, partial response (PR) was observed in 14 patients (30.4%), stable disease (SD) in 3 patients (6.5%), and progressive disease (PD) in 8 patients (17.4%); the remaining 21 patients (45.7%) were non-PR/non-PD. Esophagectomy was performed in 36 patients (78.3%), all of whom achieved R0 resection (100%). Thoracoscopic esophagectomy was performed in 22 patients (61.1%) and robot-assisted esophagectomy in 14 patients (38.9%). Lymph node dissection was D3 in 35 patients (97.2%) and D1 in 1 patient (2.8%). The pathological response rate was as follows: among the 31 patients, 20 were Grade 1 (1a: 12 patients, 33.3%; 1b: five patients, 13.9%), 10 were Grade 2, and eight were Grade 3. Pathological complete response (ypT0N0) was achieved in 5 patients (13.9%) (Table 3). The baseline distribution of clinical and pathological stages is shown in Fig. 2. Postoperative complications of Grade 2 or higher according to the Clavien-Dindo classification included pneumonia in 12 patients (33.3%), recurrent laryngeal nerve palsy in 7 patients (19.4%), anastomotic leakage in 2 patients (5.6%), and thrombosis in 2 patients (5.6%). There were no surgery-related deaths. Postoperative nivolumab therapy was administered to 2 patients (5.6%).

**Table 3:** Pathological response in the primary tumor. Pathologic complete response (ypT0N0M0) was observed in 5 patients (13.9%)

Pathological response	N = 36(%)
Grade 0: no part of tumor affected	1 (2.8)
Grade 1a	12 (33.3)
Grade 1b	5 (13.9)
Grade 2	10 (27.8)
Grade 3	8 (22.2)

grade 0: no part of tumor affected

grade 1a: very slightly effective

grade 1b: slightly effective

grade 2: moderately effective

grade 3: markedly effective

Among the 10 cases that did not undergo surgery, 3 cases underwent CRT due to refusal of surgery, 3 cases underwent CRT or palliative chemotherapy due to PD, and 3 cases were converted to best supportive care (BSC). One case died due to factors unrelated to treatment. Three cases that underwent CRT due to refusal of surgery resulted in one case with no recurrence, one case with recurrence followed by palliative chemotherapy, and one case transferred to another hospital. Among the cases that underwent CRT due to PD, one case had no recurrence, one case recurred and underwent palliative chemotherapy, and another case experienced a decline in PS during CRT and became BSC.

**Fig. 3:**
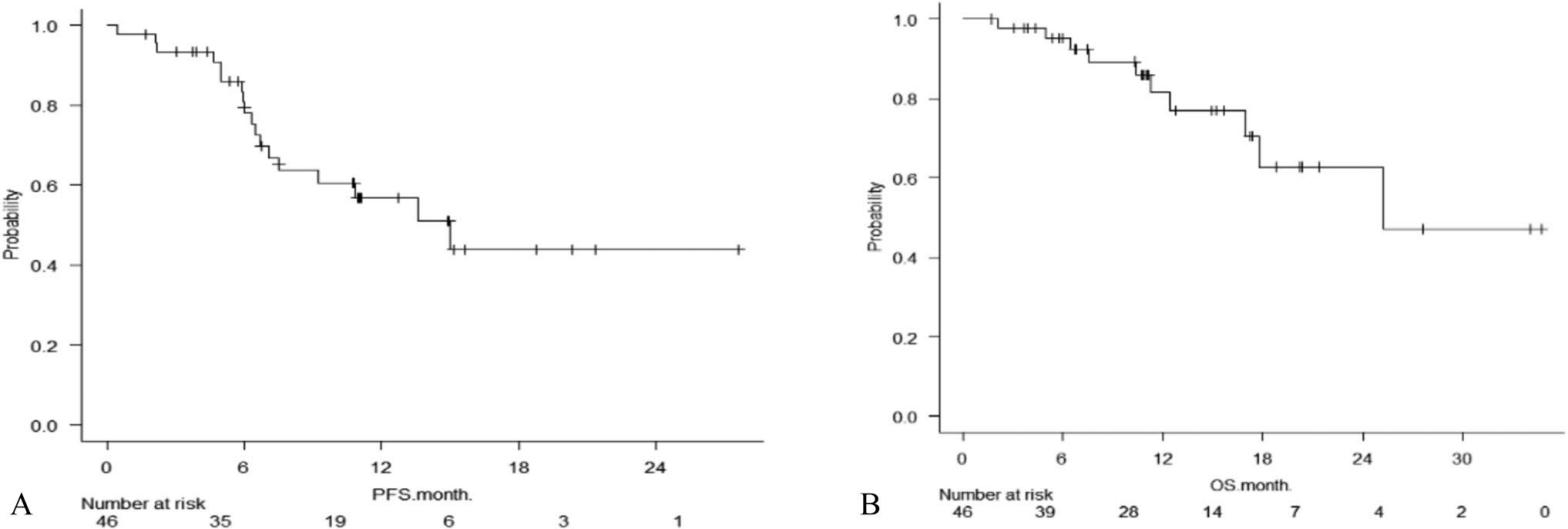
**A** Kaplan–Meier curves for progression-free survival (PFS). **B** Kaplan–Meier curves showing overall survival (OS)

The median follow-up was 10.9 months (range 1.7–34.7 months), median progression-free survival was 15.0 months (95% CI: 7.1–N.A.) (Fig. 3), and median OS was 25.2 months (95% CI: 17.0–N.A.) (Fig. 3).

## Discussion

In this study, we evaluated the neo FLOT therapy within CDDP-unfit patients with resectable LA-ESCC, resulting in a promising pathologic response with acceptable adverse events.

The pathological responses of neo FLOT in this study seemed to be comparable to these of neo DCF in the results of the JCOG1109 study [6], which have been conducted with the patients who had better condition. The pathological response (ypT0) rate grade 3 was reported at 22.2% after neo FLOT therapy in this study, comparable to 21.9% reported after neo DCF therapy [6]. In addition, pathological complete response (ypT0N0) was observed in 5 patients (13.9%). This result seemed to be comparable to the 18.6% for neo DCF therapy. A multicenter phase II trial to evaluate the efficacy of neo FLOT therapy in Japan showed pathologic response (grade 2 or 3) and grade 3 pathological response of primary lesions were 43.4% and 15% [15]. Compared to the pathological results in this study, the short-term pathological efficacy of neo FLOT therapy was comparable. Furthermore, downstaging was achieved in 72% of our patients (Fig. 2), and the R0 resection rate was 100%, which is comparable to 93.4% for neo DCF therapy. Considering these points, neo FLOT therapy have a potential to be a promising neoadjuvant therapy option for CDDP-unfit ESCC patients.

The safety profiles during neoadjuvant therapy seemed manageable. Severe neutropenia often occurred, but non-hematologic adverse events such as FN were limited. In hematological adverse events of this study, grade 3 or higher neutropenia was seen in 65.2% of patients, and FN due to neutropenia was only seen in 10.9%. This is relatively lower than the 85% of grade 3 or higher neutropenia and 16% of FN in neo DCF therapy, according to the results of the JCOG1109 study. Next, regarding non-hematological adverse events, in particular, gastrointestinal toxicity in this study was tolerable. On the other hand, neo DCF therapy is a treatment with concerns about gastrointestinal and renal toxicity due to CDDP. In this study, nausea and loss of appetite of grade 3 or higher were reported at only 2.2% and 4.3%, which was less frequent than the anorexia of neo DCF therapy in the results of the JCOG1109 study. Furthermore, in neo DCF therapy, hyponatremia was frequent, with 89% of all grades and 26% of grade 3 or higher, but in neo FLOT therapy, it was 48% of all grades, and there were no cases of grade 3 or higher.

Postoperative complications were observed more frequently after neo FLOT therapy than after neo DCF therapy, especially pneumonia and recurrent laryngeal nerve palsy (33.3% and 19.4%, respectively, after neo FLOT therapy and 9.8% and 10.4%, respectively, after neo DCF therapy). We consider that these results are due to the high proportion of elderly patients in this study and the difference between the retrospective study and the clinical trial. However, there were no in-hospital deaths.

There were several limitations of this study. First, the study was conducted retrospectively at a single institution. Therefore, it is difficult to compare the efficacy and safety between neo DCF and neo FLOT, precisely. The further investigations which compare neo DCF with neo FLOT prospectively are needed. Moreover, a comparison with FOLFOX, which is used for CDDP-unfit cases, will also be required. Second, the selection of neo FLOT in our clinical practice was based on the judgement of each medical oncologist, and the unified criteria were difficult to clarify in all patients. Although most of the patients were considered CDDP-intolerant, it is possible that the patients were affected by frailty, which is difficult to determine by renal function or ECOG PS alone. Third, survival data are immature due to short follow-up and few events. However, the pathological complete response rate is high, and further investigation of longer-term efficacy is warranted. Fourth, all patients in this study underwent mediastinoscopic or robot-assisted esophagectomy. Although these techniques may be less invasive, their impact on recurrence and survival is unknown.

In conclusion, neo FLOT therapy demonstrated manageable safety profiles and promising pathologic response in patients with resectable LA-ESCC who were unfit for CDDP-containing therapy.
